# Postexercise Recovery of Schlemm's Canal and Intraocular Pressure in Healthy Individuals: An Observational Study Using Swept-Source Optical Coherence Tomography

**DOI:** 10.1155/2018/8513760

**Published:** 2018-08-30

**Authors:** Mu Li, Xiaoqin Yan, Zhaoxia Luo, Hong Zhang

**Affiliations:** Department of Ophthalmology, Tongji Hospital, Tongji Medical College, Huazhong University of Science and Technology, Wuhan, China

## Abstract

**Purpose:**

To observe the recovery process of postexercise Schlemm's canal (SC) and intraocular pressure (IOP) in healthy individuals.

**Methods:**

Twenty healthy individuals were recruited. SC and IOP were evaluated before exercise, immediately after exercise, and 15, 30, and 60 minutes after exercise. Superior, inferior, nasal, and temporal SC quadrants were evaluated using swept-source optical coherence tomography (SS-OCT).

**Results:**

Average SC area (3726.81 ± 1167.06 vs. 4660.57 ± 1284.82 *µ*m^2^) and perimeter (324.11 ± 58.95 vs. 367.19 ± 73.34 *µ*m) increased, and IOP (14.02 ± 2.33 vs. 11.65 ± 1.90 mmHg) decreased significantly during exercise (all *p* < 0.001). After exercise, both SC and IOP recovered to preexercise values, and the recovery time for postexercise SC dimensions (15 minutes) was shorter than that for postexercise IOP (60 minutes). After adjusting for age, gender, axial length, central corneal thickness, and spherical equivalent, postexercise changes in SC dimensions were not significantly associated with postexercise changes in IOP compared with preexercise values (all *p* > 0.05). There were no significant differences in the observable SC proportion before and after exercise (all *p* > 0.05).

**Conclusions:**

The exercise-induced SC expansion and IOP reduction could recover to preexercise values after exercise, and SC recovered to preexercise values ahead of IOP. Moreover, SC might be regulated by the sympathetic nerves and could be an important causative factor of changes in IOP during and after exercise.

## 1. Introduction

In 1973, the relationship between Schlemm's canal (SC) and intraocular pressure (IOP) was reported by Johnstone and Grant [1]. They suggested that an acute elevation in IOP may result in the collapse of SC, which would in return increase the outflow resistance and form a vicious cycle to further increase IOP. Thus, the collapse of SC would increase the outflow resistance, leading to an increase in IOP [1–3] and conversely, SC is a flexible-walled lumen, and its dimensions also depend on IOP. Changes in IOP may also influence the morphology of SC [1, 4, 5].

Previous studies have indicated that aerobic exercise can induce a reduction in IOP. And after aerobic exercise, IOP can gradually increase to the preexercise level, and this postexercise IOP recovery has been suggested to take 30–60 minutes [6–8]. Furthermore, using high-frequency ultrasound biomicroscopy (UBM), a previous study found that aerobic exercise not only induced a reduction in IOP, but also an expansion of SC [9]. However, given that UBM is a contact examination method and requires surface anesthesia, repeated and multiple UBM examinations over a short period of time (e.g., 60 minutes) would injure the corneal epithelium, making the participants unable to cooperate. Thus, in the aforementioned previous study, UBM examinations were only conducted before and immediately after aerobic exercise and not further conducted at other time-points (e.g., 15, 30, and 60 minutes) after aerobic exercise to observe the recovery process of postexercise SC. Therefore, the recovery process of postexercise SC and the sequence order between the recovery of SC and IOP were still not clear.

With the advance of optical coherence tomography (OCT), the newly developed swept-source OCT (SS-OCT) has a higher scan speed and a higher axial resolution, leading to a more detailed and clear *in vivo* observation of anterior chamber angle biometrics and being a reliable SC measurement method [10, 11]. Furthermore, SS-OCT is also a noncontact and real-time examination method. Thus, it can be used to evaluate SC repeatedly (e.g., 0, 15, 30, and 60 minutes) after aerobic exercise.

The aim of this study was to investigate the recovery process of postexercise SC and its relationship with postexercise IOP recovery using SS-OCT in healthy individuals, in an effort to gain a better understanding of SC and its relationship with IOP.

## 2. Materials and Methods

This study was approved by the ethics committee of Tongji Hospital and adhered to the tenets of the Declaration of Helsinki. All subjects provided written informed consent prior to participation in the study.

### 2.1. Subjects

Forty eyes from twenty healthy young individuals were included. They underwent measurements of best-corrected visual acuity (BCVA), refractive error, central corneal thickness (CCT), axial length (AL), slit-lamp examination, gonioscopy, visual field test, and fundus photography. The inclusion criteria were as follows: age ≥ 18 years; IOP of 10–21 mmHg (Goldmann tonometry); normal visual field; and no use of drugs affecting the circulatory system within a month prior to evaluation. Subjects with unclear ocular media, family history of glaucoma, medical history of ophthalmic diseases or surgery, or systemic diseases and low-quality OCT images (evaluated on the basis of visibility of scleral spurs and iris, continuity of anterior chamber structures, and motion artifacts) were excluded. Both eyes of each subject were selected for IOP and SS-OCT examinations, and all the examinations were performed in a right-to-left eye order [12]. Participants were requested to abstain from alcohol and caffeine for 3 days and also not to consume any food or beverage for at least 30 minutes before participating in this study.

#### 2.1.1. IOP, Blood Pressure, and Heart Rate Measurements and Control of Exercise Intensity

The participants rested for 20 minutes and then performed aerobic exercise by jogging on the treadmill for 20 minutes. After jogging, they were instructed to rest (sitting quietly) for another 60 minutes. IOP and blood pressure (BP) were measured just before exercise (to evaluate the status at rest), at 0 minute (immediately) after exercise (to evaluate the status during exercise) and at 15, 30, and 60 minutes after exercise (to evaluate the status after exercise) by the same operator and instruments. IOPs were measured using a noncontact tonometer (NIDEK RT-2100; NIDEK, CO., LTD, Gamagori, Japan). Three measurements were obtained, and the average IOP was recorded. BPs were recorded using an automatic sphygmomanometer (OmronHEM-7201; Omron, Dalian, China). Heart rate (HR) was monitored throughout the exercise with an oximeter (prince-100B; Heal Force, CO., LTD, Shanghai, China) for controlling exercise intensity (aerobic exercise) by evaluating the percentage of heart-rate reserve (% HR_max_) [13].

#### 2.1.2. SS-OCT Imaging Acquisition and Processing

SS-OCT (CASIA SS-1000; Tomey Corp., Nagoya, Japan) has a 1310 nm wavelength with a scan speed of 30,000 A-scans/s and an axial resolution of less than 10 *µ*m. The participants were imaged with a high-density scan (HD, a raster scan covering an area of 8 × 4 mm^2^ for an individual quadrant; 64 B-scans each with 512 A-scans of 8 mm) and a low-density scan (LD, a radial scan covering the whole anterior chamber for 360 degrees; 128 radial scans each with 512 A-scans of 16 mm) [10]. The scans were performed in each eye just before and at 0, 15, 30, and 60 minutes after exercise in the dark room. The participants were instructed to open the eye wide during examination, and if necessary, the examiner would lightly pull the participants upper or lower eyelid to expose the scan area adequately and avoid pressing the eye, to ensure accurate measurement of SC. With the HD protocol, the superior, nasal, inferior, and temporal limbi were recorded separately after adjusting the fixture to the corresponding areas. With the LD protocol, the entire anterior chamber was recorded.

The scans were performed three times, and the best-quality image was chosen for analysis. SC was defined as observable when a thin, black, lucent space was detected in two or more consecutive horizontal B-scan images [14]. Optimum image contrast and magnification were subjectively defined in order to maximize the visualization of the SC. The dimensions (area and perimeter) of SC were manually drawn freehand based on the outline of SC using the ImageJ software (National Institutes of Health, Bethesda, Maryland, USA) [9, 15] by two separate experienced operators (ML and ZL), who were masked to the subject information. The percentage of observable SC was calculated as follows: eyes with observable SC/total number of eyes × 100%. Pupil diameter was measured as the distance from one side of the pupillary tip of the iris to the opposite side on the LD images.

### 2.2. Blood Sample Test

Blood samples were collected by each subject before and at 0, 15, 30, and 60 minutes after exercise, in vacuum with EDTA. Plasma was obtained by centrifugation of blood sample at 1000 × g (Heraeus Multifuge X1R; Thermo Scientifc, Osterode, Lower Saxony, Germany) for 10 minutes at 4°C and stored at −80°C. Plasma noradrenaline (NA) and adrenaline (A) concentration were tested using high-performance liquid chromatography (HPLC) with electrochemical detectors (Waters HPLC pump, model 515; Waters electrochemical detector, model 2465; Waters autosampler, model 717; Atlantic C18 column (4.6 mm × 150 mm); Waters, Milford, MA, USA).

### 2.3. Statistical Analysis

All analyses were performed using the SPSS software package version 21.0 (IBM Corp., Armonk, NY, USA). Data were presented as mean ± standard deviation (SD) where applicable. The generalized estimate equations, which take into account the correlation between the measurements from two eyes of one subject, were performed to compare pupil diameter, SC and IOP before and (0, 15, 30, 60 minutes) after exercise, and linear mixed-effects models were performed to compare blood pressure and plasma catecholamine before and (0, 15, 30, 60 minutes) after exercise. After age, sex, axial length, central corneal thickness and spherical equivalent were adjusted, univariate regression analysis was used to quantify the associations of postexercise changes in SC dimensions with postexercise changes in IOP. Univariable-adjusted *β* coefficients, with 95% confidence intervals (CIs), for the associations between independent and dependent variables were assessed using generalized estimating equations. The Chi-square test was used to compare the observable SC proportions before and after exercise. The interobserver agreement was assessed with the intraclass correlation coefficient (ICC). All tests were two-tailed, and statistical significance was defined as a *p* value less than 0.05.

## 3. Results

### 3.1. Subject Characteristics

A total of 40 eyes of 20 subjects (9 males; 11 females; mean age was 26 ± 3 years) were included. Mean CCT was 540 ± 29 *μ*m, mean AL was 25.20 ± 1.04 mm, and mean refraction was −3.64 ± 1.97 diopters. The mean percent maximal heart rate (%HR_max_) during exercise was 69.04 ± 3.58%.

### 3.2. Comparison of Blood Pressure, Plasma Catecholamine, Pupil Diameter, Schlemm's Canal, and IOP before and after Exercise

**Figure 1:**
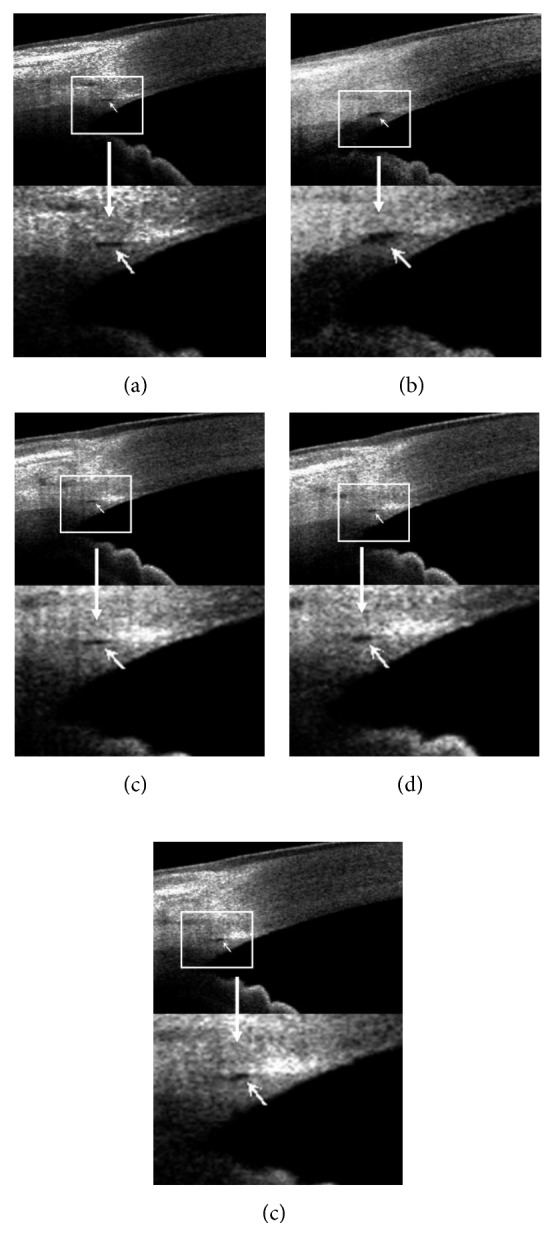
Schlemm's canal before exercise and 0 minutes (immediately), 15 minutes, 30 minutes, and 60 minutes after exercise: the white arrow indicated Schlemm's canal. (a) Before exercise, (b) 0 minute after exercise, (c) 15 minutes after exercise, (d) 30 minutes after exercise, and (e) 60 minutes after exercise.

Compared with preexercise (baseline) values, systolic blood pressure (SBP), diastolic blood pressure (DBP), plasma catecholamine (noradrenaline (NA), adrenaline (A)), and pupil diameter (PD) increased significantly during exercise (*β* = 32.75 [29.61, 35.89], 7.90 [5.31, 10.49], 6.11 [4.77, 7.45], 0.70 [0.50, 0.90], and 0.32 [0.15, 0.49], respectively; all *p* < 0.001). After 15 minutes of recovery, SBP, DBP, plasma catecholamine (NA and A), and PD reduced to the baseline (*β* = 2.40 [−0.75, 5.54], 1.55 [−1.04, 4.14], 0.27 [−1.07, 1.61], −0.05 [−0.25, 0.15], and 0.09 [−0.02, 0.20], respectively; *p* = 0.139, 0.245, 0.691, 0.620, and 0.098, respectively). Similar to BP, plasma catecholamine, and PD, the average SC area and perimeter increased significantly during exercise (*β* = 933.76 [572.71, 1294.82] and 43.08 [22.46, 63.71], respectively; both *p* < 0.001) and also recovered to preexercise values (*β* = 57.41 [−297.13, 411.95] and 4.85 [−15.23, 24.92], respectively; *p*=0.751  and  0.636, respectively) within 15 minutes. Moreover, IOP decreased significantly during exercise (*β* = −2.37 [−2.71, −2.03]; *p* < 0.001) and recovered to preexercise value (*β* = 0.02 [−0.31, 0.34]; *p*=0.927) within 60 minutes. The recovery time for postexercise SC was shorter than that of postexercise IOP and postexercise SC recovered ahead of postexercise IOP (Table 1 and Figure 1).

### 3.3. Associations of Postexercise Changes in Schlemm's Canal with Postexercise Changes in IOP Compared with Preexercise (Baseline) Values

After adjusting for age, sex, axial length, central corneal thickness, and spherical equivalent, the univariate regression analysis results showed that compared with preexercise (baseline) values, changes in SC area and perimeter were not significantly associated with changes in IOP at any postexercise measurement time-points (all *p* > 0.05) (Table 2).

### 3.4. Comparison of Observable Schlemm's Canal Proportion before and after Exercise

There were no significant differences in the observable SC proportion before and after exercise in any quadrant, or in total (all *p* > 0.05) (Table 3).

### 3.5. Interobserver Agreement of Schlemm's Canal Dimensions Measurements

Our results showed a good interobserver agreement of SC dimensions measurements before exercise, 0 minute after exercise, and at 15, 30, and 60 minutes after exercise (Table 4).

## 4. Discussion

Previous studies have demonstrated that SC and IOP are inversely correlated [1–5, 15]. In terms of exercise, aerobic exercise can induce expansion of SC with a reduction in IOP [9]. However, the postexercise SC recovery and its relationship with postexercise IOP recovery have not been determined for now. In the present study, using SS-OCT, a noncontact and high resolution examination method, we successfully observed the postexercise SC recovery and found that the expanded postexercise SC reduced back to its baseline dimensions within 15 minutes, while the reduced postexercise IOP increased back to baseline within 60 minutes. Thus, both during and after exercise, changes in SC were the inverse of changes in IOP (SC dimensions increased with IOP reduced during exercise and vice versa after exercise). However, we also observed that although both postexercise SC and IOP recovered from exercise, their recovery processes were not simultaneous, but had a sequence order. The SC recovery time was shorter than the IOP recovery time, and SC recovered to its baseline ahead of IOP. Therefore, we speculated that postexercise SC reduced its dimensions to baseline first, and then the aqueous humor outflow facility decreased in accordance with the SC collapse [1], finally resulting in an increase in IOP back to baseline. Changes in SC dimensions might be an important causative factor of changes in IOP.

With regard to the reasons for changes in SC during and after exercise, autonomic nerves may play an important role. Previous studies have found that *β*2-adrenergic receptors have been detected in SC [16]. Alvarado et al. administered adrenaline and isoproterenol to reduce the size of SC endothelial cells, enlarge the intercellular space, and increase the outflow facility via activating *β*2-adrenergic receptors [17]. Zhou et al. also observed that isoproterenol could decrease SC endothelial cell stiffness and increase SC endothelial cell compliance due to its effect on *β*2-adrenergic receptors; hence, the resistance of aqueous outflow was resolved; moreover, they also found that the expression level of *β*2-adrenergic receptors is positively correlated with the degree of relaxation triggered by isoproterenol [16]. These results suggest that SC may be regulated by the sympathetic nervous system [9].

In the current study, BP and PD increased significantly during exercise, indicating that exercise intensity of our study participants was sufficient to activate the sympathetic nervous system [18–22]. And after exercise, both BP and PD reduced to preexercise values within 15 minutes. Moreover, the current study and prior studies [23, 24] demonstrated that plasma catecholamine, an indicator of sympathetic nervous activity [25], also increased during exercise and recovered to normal range within 15 minutes after exercise. Thus, the findings in this study suggested that exercise could activate the sympathetic nervous system, and after exercise, the sympathetic nervous system activity would decrease back to normal within 15 minutes. In terms of SC, its dimensions increased during exercise, and the recovery time of postexercise SC was also within 15 minutes, as is the case for BP, PD, and catecholamine. The change in SC and the changes in BP, PD, and catecholamine exhibit good time consistency both during and after exercise. Thus, we speculated that during exercise, SC expanded with an increase in sympathetic nervous activity, leading to a reduction in IOP, and after exercise, SC collapsed with a decrease in sympathetic nervous activity. After SC collapsed to its baseline dimensions, the aqueous humor outflow facility would also decrease, resulting in a gradual elevation in IOP.

However, after adjusting for age, sex, axial length, central corneal thickness, and spherical equivalent, postexercise changes in SC dimensions were not significantly associated with postexercise changes in IOP. With regard to IOP, there are also factors other than SC that may contribute to changes in IOP during and after exercise, including changes in aqueous humor production and changes in the trabecular outflow facility, which were caused by exercise-induced changes in ocular blood supply, colloidal osmotic pressure of plasma, and catecholamine concentration [22, 26–29]. Therefore, changes in IOP might be caused by multiple factors and could not be solely explained by SC. Moreover, with regard to SC, its endothelium cells are able to contract [16], and SC might have autonomic regulation. Thus, its expansion and collapse may not be fully dependent on IOP [9].

In the present study, we only observed a slight, nonsignificant increase in the observable SC proportions between 0 minutes (immediately) after exercise and other time-points, indicating that exercise-induced changes in SC may mainly occur in SC dimensions, not the observable SC proportion.

This study has certain limitations. First, we only recruited healthy individuals but no patients with glaucoma. Considering that previous studies have suggested that exercise might have a long-term effect on the reduction in IOP and contributed to the lower baseline IOP, indicating a role of exercise in the glaucoma prevention and management [29–32], further research is needed to investigate the postexercise changes and recoveries of SC and IOP in glaucomatous groups. Second, all the participants in this study were young myopic individuals, and it is unclear whether similar results could be observed in elderly group and emmetropic or hypermetropic groups.

In conclusion, exercise induced expansion of SC and reduction in IOP. Both of these changes recovered after exercise. SC returned to baseline values first (within 15 minutes), followed by IOP (within 60 minutes). Moreover, SC may be regulated by sympathetic nerves. Thus, during exercise, the activation of sympathetic nerves may increase SC dimensions and result in the reduction in IOP. After exercise, the sympathetic nerve activity decreased, leading to the reduction in SC dimensions. The decrease in SC dimensions reduced the aqueous humor outflow facility and finally caused the elevation in IOP.

## Figures and Tables

**Table 1:** Comparison of BP, plasma catecholamine, PD, SC, and IOP before and after exercise.

	Before exercise (baseline)	0 min after exercise	15 min after exercise	30 min after exercise	60 min after exercise
SBP (mmHg)	110.7 ± 8.4	143.5 ± 10.2^†^	113.1 ± 9.1	112.1 ± 12.1	108.7 ± 9.3
DBP (mmHg)	75.3 ± 7.5	83.2 ± 7.6^†^	76.8 ± 8.1	75.5 ± 5.7	76.0 ± 4.5
NA (nmol/L)	3.15 ± 1.23	9.26 ± 5.46^†^	3.42 ± 1.52	3.24 ± 1.36	3.31 ± 1.18
A (nmol/L)	0.49 ± 0.28	1.18 ± 0.90^†^	0.43 ± 0.23	0.45 ± 0.22	0.46 ± 0.28
PD (mm)	4.50 ± 0.67	4.81 ± 0.94^‡^	4.58 ± 0.78	4.54 ± 0.87	4.56 ± 0.72

SC area (*µ*m^2^)					
Superior	4056.03 ± 1608.33	4894.49 ± 1783.57^‡^	3990.63 ± 1967.99	3936.21 ± 1371.07	3958.88 ± 1333.92
Nasal	3245.64 ± 1229.18	4086.96 ± 1612.77^‡^	3376.63 ± 1106.34	3349.13 ± 979.86	3219.68 ± 1087.43
Inferior	3936.06 ± 1774.87	4802.46 ± 1697.76^‡^	3911.81 ± 1438.14	3854.22 ± 1445.20	4252.15 ± 1861.88
Temporal	4015.16 ± 2199.23	5002.51 ± 2386.53^‡^	4254.10 ± 2015.28	3544.64 ± 1645.59	3751.98 ± 1454.45
Average	3726.81 ± 1167.06	4660.57 ± 1284.82^‡^	3784.22 ± 1193.19	3598.93 ± 937.01	3752.92 ± 951.11

SC perimeter (*µ*m)					
Superior	347.79 ± 105.80	390.26 ± 126.17^‡^	342.82 ± 121.52	355.85 ± 103.59	357.08 ± 109.34
Nasal	293.57 ± 69.02	330.20 ± 99.41	312.75 ± 82.78	320.06 ± 70.52	297.08 ± 72.63
Inferior	320.27 ± 92.93	358.28 ± 96.55^‡^	318.16 ± 84.38	304.96 ± 79.77	333.45 ± 96.27
Temporal	346.13 ± 107.83	393.32 ± 127.93^‡^	356.83 ± 122.54	336.62 ± 111.07	333.91 ± 70.83
Average	324.11 ± 58.95	367.19 ± 73.34^‡^	328.96 ± 72.31	323.92 ± 61.83	328.26 ± 48.38

IOP (mmHg)					
Average	14.02 ± 2.33	11.65 ± 1.90^‡^	13.16 ± 2.26^‡^	13.49 ± 2.35^‡^	14.04 ± 2.30

^†^Significance of difference between preexercise (baseline) and (0, 15, 30, and 60 min) postexercise data: linear mix-effect models. ^‡^Significance of difference between preexercise (baseline) and (0, 15, 30, and 60 min) postexercise data: general estimate equations. SBP: systolic blood pressure, DBP: diastolic blood pressure, NA: noradrenaline, A: adrenaline, PD: pupil diameter, SC: Schlemm's canal, IOP: intraocular pressure.

**Table 2:** Univariate regression analysis of the associations of postexercise changes in SC dimensions with postexercise changes in IOP compared with preexercise (baseline) values.

	Before vs. 0 min after exercise		Before vs. 15 min after exercise		Before vs. 30 min after exercise		Before vs. 60 min after exercise	
	ΔSC area (*µ*m^2^)		ΔSC area (*µ*m^2^)		ΔSC area (*µ*m^2^)		ΔSC area (*µ*m^2^)	
ΔIOP (mmHg)	*β* [95% CI]	*p*	*β* [95% CI]	*p*	*β* [95% CI]	*p*	*β* [95% CI]	*p*
	−223.19 [−501.79, 55.40]	0.116	−276.49 [−602.93, 50.00]	0.097	−216.31 [−466.28, 33.67]	0.090	−232.94 [−520.05, 54.18],	0.112

	ΔSC perimeter (*µ*m)		ΔSC perimeter (*µ*m)		ΔSC perimeter (*µ*m)		ΔSC perimeter (*µ*m)	
ΔIOP (mmHg)	*β* [95% CI]	*p*	*β* [95% CI]	*p*	*β* [95% CI]	*p*	*β* [95% CI]	*p*
	−10.98 [−24.41, 2.46]	0.109	−15.92 [−37.37, 5.53]	0.146	−4.52 [−24.78, 15.74]	0.662	−3.99 [−22.38, 14.40]	0.671

*β*/*P* value: regression coefficient and *p* values of the independent variables in the generalized estimating equations. The influence factors as age, sex, axial length, central corneal thickness, and spherical equivalent have been adjusted. Δ, the change of the variables between 0, 15, 30, and 60 minutes postexercise and preexercise (baseline) values. IOP: intraocular pressure, SC: Schlemm's canal, CI: confidence interval.

**Table 3:** Comparison of observable Schlemm's canal proportion before and after exercise.

Observable SC proportion	Before exercise (baseline)	0 min after exercise	15 min after exercise	30 min after exercise	60 min after exercise	*X*	*p*
Superior quadrant	34/40	37/40	34/40	33/40	33/40	2.178	0.703
Nasal quadrant	34/40	37/40	36/40	35/40	33/40	2.286	0.683
Inferior quadrant	32/40	36/40	34/40	36/40	33/40	2.581	0.630
Temporal quadrant	38/40	39/40	35/40	36/40	35/40	4.243	0.374
In total	138/160	149/160	139/160	140/160	134/160	6.971	0.137

SC: Schlemm's canal.

**Table 4:** Interobserver agreement of SC dimensions measurements.

	ICC	Difference	95% CI	
			Lower	Upper
Average SC area (*µ*m^2^)				
Before exercise	0.902	189.83	0.823	0.947
0 min after exercise	0.923	229.34	0.859	0.958
15 min after exercise	0.896	102.12	0.811	0.943
30 min after exercise	0.880	14.82	0.784	0.935
60 min after exercise	0.893	55.09	0.807	0.942

Average SC perimeter (*µ*m)				
Before exercise	0.874	1.63	0.774	0.931
0 min after exercise	0.934	5.04	0.879	0.965
15 min after exercise	0.897	1.68	0.814	0.944
30 min after exercise	0.896	2.93	0.812	0.943
60 min after exercise	0.895	10.54	0.810	0.943

SC: Schlemm's canal; ICC: intraclass correlation coefficient; CI: confidence interval.

## Data Availability

The readers could access the data related to this manuscript by contacting the corresponding author out of reasonable requests.
